# Work-related violence from a gender perspective: a cross-sectional study with temporal trend analysis, Bahia, Brazil, 2009-2024

**DOI:** 10.1590/S2237-96222026v35e20251061.en

**Published:** 2026-08-18

**Authors:** Adriana Galdino, Anívia de Souza Amaral, Érika Maria Andrade Silva, Alice Leite Velôso, Tarsila Rios Cabral, Ive Caroline Almeida Xavier

**Affiliations:** 1 Universidade Estadual do Sudoeste da Bahia, Campus de Jequié, Jequié, BA, Brazil Universidade Estadual do Sudoeste da Bahia Universidade Estadual do Sudoeste da Bahia Campus de Jequié Jequié BA Brazil; 2 Secretaria Municipal de Saúde de Jequié, Jequié, BA, Brazil Secretaria Municipal de Saúde de Jequié Secretaria Municipal de Saúde de Jequié Jequié BA Brazil

**Keywords:** Workplace Violence, Gender Perspective, Health Information Systems, Gender-Based Violence, Occupational Health, Violencia Laboral, Perspectiva de Género, Sistemas de Información en Salud, Violencia de Género, Salud Laboral.

## Abstract

**Objective:**

To analyze cases of work-related violence from a gender perspective in Bahia state, Brazil, from 2009 to 2024, considering sociodemographic characteristics and violent events.

**Methods:**

A descriptive, cross-sectional study with temporal trend analysis was conducted using data on work-related violence obtained from the Notifiable Health Conditions Information System, including individuals aged ≥18 years. Relative frequencies, proportional distributions, bivariate analyses between descriptive variables and sex (female and male), and temporal trend analysis of the case reporting rate were estimated.

**Results:**

A total of 1,768 cases of work-related violence were recorded in Bahia during the period. A higher frequency was observed among women (55.8%). For women, violence was more frequent among individuals aged 18-29 years (36.5%) and among those of mixed race (46.7%). Cases were also motivated by sexism (9.7%) and occurred in the home setting (27.9%). For males, it predominated in the same age group (35.0%) and among those of mixed race (49.2%), in addition to being motivated by generational conflict (5.9%) and occurring in public thoroughfares (27.3%). Trend analysis showed an increasing reporting rate for both sexes, with 15% annual percentage change (95% confidence interval, 95%CI 15.01;15.09) in women and 15.1% (95%CI 15.11;15.19) in men.

**Conclusion:**

Women disproportionately experience work-related violence in Bahia, highlighting the urgency of addressing this phenomenon as a structural and gender-based problem. The development of effective policies for preventing and combating this type of violence is fundamental.

## Resumen

**Objetivo:**

Analizar casos de violencia laboral desde una perspectiva de género en Bahía, Brasil, entre 2009 y 2024, considerando aspectos sociodemográficos y eventos de violencia.

**Métodos:**

Se realizó un estudio descriptivo transversal con análisis de tendencias temporales utilizando datos sobre violencia laboral obtenidos del Sistema Nacional de Información sobre Enfermedades de Declaración Obligatoria, que involucran a personas mayores de 18 años. Se estimaron frecuencias relativas, distribuciones proporcionales, análisis bivariados entre variables descriptivas y sexo (femenino y masculino), y análisis de tendencias temporales de la tasa de notificación de casos.

**Resultados:**

Se registraron 1768 casos de violencia laboral en Bahía durante el período. Se observó una mayor frecuencia entre las mujeres (55,8%). En el caso de las mujeres, la violencia predominó en el grupo de edad de 18 a 29 años (36,5%) y entre aquellas de raza mixta (46,7%). También estuvo motivada por el sexismo (9,7 %) y ocurrió en el hogar (27,9 %). En los hombres, predominó en el mismo grupo de edad (35,0 %) y entre los mestizos (49,2 %), además de estar motivada por el conflicto generacional (5,9 %) y ocurrir en vía pública (27,3 %). El análisis de tendencias reveló un aumento en la tasa de notificación en ambos sexos, con un cambio porcentual anual del 15,0 % (intervalo de confianza del 95 %, IC95% 15,01; 15,09) en mujeres y del 15,1 % (IC95% 15,11; 15,19) en hombres.

**Conclusión:**

Las mujeres sufren desproporcionadamente más violencia laboral en Bahía, lo que subraya la urgencia de abordar este fenómeno como un problema estructural y de género. El desarrollo de políticas efectivas para la prevención y el combate de este tipo de violencia es fundamental.

Ethical aspects This research used anonymized, public-domain databases.

## Introduction

Workplace violence is defined as a set of behaviors, practices, or threats, occurring one or more times, that cause or intend to cause physical, psychological, sexual, or economic harm [1]. Workplace harassment is an example of violence characterized by systematic actions of humiliation and embarrassment practiced repeatedly [2]. Regarding the impact of these phenomena, the International Labor Organization, in its first global survey on violence and harassment at work, revealed that more than one in five workers have been affected by these forms of abuse during their working lives [3].

Gender-based violence and harassment in the workplace are manifestations of occupational violence and, in this study, are also treated as work-related violence. Gender issues permeate labour markets, since, being understood as a social construct based on perceived differences between the sexes, this category structures power relations that hierarchize men to the detriment of women in different contexts [4]. In different spheres of the occupational environment, opportunity and wage inequalities can be observed, in addition to situations of moral and sexual harassment and gender-related violence [5].

Power relations in the workplace are complex. Aggressions do not occur in isolation, but intersect with other social markers, such as class, race/ color, ethnicity, disability and religion [6]. Women experiencing discrimination in public and private life tend to be more exposed to violence and harassment at work [3], thereby intensifying experiences of oppression and exclusion.

Work-related violence needs to be understood by considering gender implications, as it disproportionately impacts women, which can be seen in different work activities. In Iceland, data from a national cohort of women showed that, in 2019, 75% (n=1,178) experienced sexual harassment or violence in the workplace at some point in their lives [7]. In Addis Ababa, the capital of Ethiopia, among 827 female construction workers, the prevalence of workplace violence against women was 71% in 2021 [8].

Regarding the health sector in Brazil, in Diamantina, state of Minas Gerais, in 2017, prevalence of workplace violence against women was 84% among health department workers [9]. Among nurses in hospitals in Campo Grande, state of Mato Grosso do Sul, it was found that in the period 2018-2019 women suffered 5.83 times more verbal violence than men [10]. In a hospital in Itaituba, state of Pará, in 2021, 69% of female health workers in urgent and emergency care service suffered work-related violence [11]. In a university hospital in Rio Grande do Norte state, during the COVID-19 pandemic in 2020, 80% of female multi-professional residents reported physical/verbal violence [12].

In Brazil, violence is a significant public health problem, with interpersonal and self-inflicted violence being a universally reportable condition. As such, it must be reported by healthcare professionals who provide care to people experiencing violence, regardless of the type of health service facility [13]. However, research on gender-based workplace violence is limited, especially in regional contexts and with large population proportions. In this context, this study contributes to deepening the debate on workplace violence and can support the formulation of public policies and institutional strategies to address it. The objective of this study was to analyze cases of work-related violence from a gender perspective in the state of Bahia, from 2009 to 2024, considering sociodemographic aspects and events of violence.

## Methods

### Design and setting

This is a descriptive, cross-sectional study with temporal trend analysis, in which records of work-related violence were analyzed from a gender perspective. In this study, the gender perspective was defined as the presentation and analysis of data stratified by sex (female and male). We assessed reported cases of work-related violence among individuals aged ≥18 years, residing in municipalities in the state of Bahia, occurring between 2009 and 2024, this being the period for which data was available for analysis. Bahia has an estimated population of 14,141,626 inhabitants according to the 2022 census, of which 7,305,940 are women (51.7%). The state comprises 417 municipalities, distributed across nine Health Regions.

This study was conducted as part of the Education for Work in Health Program (*Programa de Educação para o Trabalho pela Saúde, PET-Saúde*), funded by the Brazilian Ministry of Health. The 11th edition of the Program addressed the theme of Equity, with its structuring axis focusing on the Valorization of current and future female workers within the Brazilian Unified Health System, mental health, and work-related violence in healthcare. The researchers in this study are part of this edition of PET, which, on this occasion, was carried out through a partnership between the State University of Southwest Bahia (*Universidade Estadual do Sudoeste da Bahia*) and the Municipal Health Department of Jequié, Bahia.

### Data sources

Violence records were obtained from the Notifiable Health Conditions Information System (*Sistema de Informação de Agravos de Notificação, SINAN*), using the Interpersonal and Self-Inflicted Violence Investigation Form. This instrument is used nationwide to report cases of domestic/intrafamily, sexual and self-inflicted violence, as well as human trafficking, forced labor, child labor, torture, legal intervention and homophobic violence, including women and men of all ages in Brazil [13]. Population data were also obtained from the Brazilian Institute of Geography and Statistics portal for Bahia, by sex and year.

### Eligibility criteria

The Interpersonal and Self-Inflicted Violence Investigation Form has 69 fields with information ranging from victim identification to referrals. In the database provided to us, in all cases the “work-related violence” field was recorded as “1=yes”, totaling 2,328 observations. Of these, 557 cases were excluded due to the victim being under 18 years of age and three due to unknown sex records. No imputations or duplicate checks were performed, as the database analyzed belonged to the Epidemiological Surveillance Directorate of the Bahia State Health Department, considering that database cleaning is routinely performed by the sector and was carried out beforehand.

### Variables

The variables were selected based on their descriptive relevance,grouped into subdomains:

1. sociodemographic variables: • sex (female; male);
  • age group (in years: 18-29; 30-39; 40-49;
  • 50-59; 60-69; >70);
  • race/color (Asian; White; Indigenous; mixed-race; Black; unknown/left blank);
  • schooling (illiterate; elementary; high school; higher education; unknown/not applicable; left blank);
  • marital status (married/cohabiting; single; widowed; separated; unknown/not applicable; left blank);
  • disability (yes; no; unknown; left blank);
  • sexual orientation (heterosexual; homosexual; bisexual; unknown/not applicable; left blank);
  • gender identity (transgender man; transgender woman; transvestite (*travesti*); unknown/not applicable; left blank);
  • pregnancy (yes; no; unknown/not applicable);
2. violence variables: • type (physical; psychological/moral; torture; sexual; human trafficking; financial/ economic; neglect/abandonment; legal intervention; other);
  • motivation (generational conflict; disability; homophobia/lesbophobia/biphobia/ transphobia; religious intolerance; racism; sexism; homelessness; xenophobia; other; unknown; none of the above);
  • place (bar or similar; commerce/services; school; shared housing; industries/ construction; sports facility; home; public thoroughfare; other; unknown; none of the above);
3. violence perpetrator variables: • relationship/degree of kinship (mother/ father; stepmother/stepfather; spouse; ex-spouse; boyfriend; ex-boyfriend; sibling/son/ daughter; stranger; acquaintance; caregiver; boss; police officer/law enforcement agent; person with an institutional relationship; self-inflicted; other; left blank);
  • sex of the probable perpetrator (female; male; both; unknown; none of the above).

Overall, categories were maintained according to the original form, except for schooling (grouped categories) and type of violence, whereby “child labor” was considered inconsistent for the age group and child labor records were aggregated in the “other” category. For all variables, “not applicable” was incorporated into the “unknown” category, as they indicated a lack of valid information.

During the analyzed period, changes were made to the fields of the form; however, these changes were considered methodologically irrelevant for the cross-sectional analysis.

### Statistical analysis

Descriptive data were presented as absolute numbers and proportions, stratified by victim sex. Work-related violence case reporting rates were estimated by year of occurrence, calculated as the ratio between the number of cases and their respective populations. Association between categorical variables was analyzed using Pearson’s chi-square test. Prais-Winsten regression was used for temporal trends, correcting for first-order autocorrelation. Reporting rates were log-transformed, considering the year as the independent variable. Annual percentage change (APC) and respective 95% confidence intervals (95%CI) were then calculated. The trend was classified as increasing when the APC 95%CI was positive; falling when negative; and stationary when it included zero. A 5% significance level was adopted in all inferential analyses. The data were analyzed using R software version 4.3.2.

## Results

Between 2009 and 2024, a total of 1,768 cases of work-related violence against people over 18 years of age were recorded in Bahia. Of these, 987 (55.8%) were against women. The highest number of cases occurred among individuals aged 18-29 years, both for females (36.5%) and males (35.0%). Regarding race/color, mixed-race individuals accounted for the largest proportion of cases, both in the case of women (46.7%) and men (49.2%). In terms of schooling, women with high school education predominated (24.5%), followed by men with elementary education (22.8%). Regarding marital status, single women were the main victims (44.4%); among men, the most common marital status was “unknown” (36.4%), followed by single (32.1%). Most cases occurred among individuals without disabilities, both in the case of women (71.2%) and men (60.1%). Regarding disability status, mental disorder was the most frequently reported category, accounting for 3.4% in females and 2.6% in males. Regarding sexual orientation, heterosexual was the most reported in female cases (48.2%); in males, the most frequent category was “unknown/not applicable” (41.9%), followed by heterosexual (38.0%). Regarding gender identity, for both sexes the “unknown/not applicable” category was the most frequent, being 77.2% for women and 80.3% for men. Most women were not pregnant (54.3%). In the bivariate analysis, all sociodemographic characteristics showed statistical significance for the relationship with the sex of victims of work-related violence (Table 1).

**Table 1:** Frequency (n) and percentage (%) of reported cases of work-related violence according to sex and sociodemographic variables. Bahia, 2009-2024 (n=1,768)

Variables	Female	Male	Total	p-value
	(n=987; 55.8%)	(n=781; 44.2%)	(n=1,768; 100%)	
	n (%)	n (%)	n (%)	
**Age group (years)**				<0.001
18-29	360 (36.5)	273 (35.0)	633 (35.8)	
30-39	315 (31.9)	198 (25.3)	513 (29.0)	
40-49	208 (21.1)	166 (21.3)	374 (21.2)	
50-59	75 (7.6)	79 (10.1)	154 (8.7)	
60-69	18 (1.8)	47 (6.0)	65 (3.7)	
>70	11 (1.1)	18 (2.3)	29 (1.6)	
**Race/color**				<0.001
Asian	9 (0.9)	43 (5.5)	52 (2.9)	
White	120 (12.2)	70 (9.0)	190 (10.7)	
Indigenous	3 (0.3)	2 (0.3)	5 (0.3)	
Mixed-race	461(46.7)	384 (49.2)	845 (47.8)	
Black	200 (20.3)	127 (16.2)	327 (18.5)	
Unknown/left blank	194 (19.6)	155 (19.8)	349 (19.8)	
**Schooling**				<0.001
Illiterate	7 (0.7)	16 (2.0)	23 (1.3)	
Elementary	168 (17.0)	178 (22.8)	346 (19.6)	
High school	237 (24.0)	107 (13.7)	344 (19.5)	
Higher education	159 (16.2)	37 (4.8)	196 (11.0)	
Unknown/not applicable	339 (34.3)	388 (49.7)	727 (41.1)	
Left blank	77 (7.8)	55 (7.0)	132 (7.5)	
**Marital status**				<0.001
Married/cohabiting	266 (27.0)	209 (26.8)	475 (26.9)	
Single	438 (44.4)	251 (32.1)	689 (39.0)	
Widowed	20 (2.0)	7 (0.9)	27 (1.5)	
Separated	39 (4.0)	17 (2.1)	56 (3.2)	
Unknown/not applicable	193 (19.5)	284 (36.4)	477 (26.9)	
Left blank	31 (3.1)	13 (1.7)	44 (2.5)	
**Disability**				<0.001
Yes	79 (8.0)	38 (4.9)	117 (6.6)	
Mental disability	16 (1.6)	5 (0.6)	21 (1.2)	
Mental disorder	34 (3.4)	20 (2.6)	54 (3.1)	
Behavior disorder	14 (1.4)	8 (1.0)	22 (1.2)	
Visual impairment	4 (0.4)	3 (0.4)	7 (0.4)	
Hearing impairment	4 (0.4)	1(0.1)	5 (0.3)	
Other	4 (0.4)	3 (0.4)	7 (0.4)	
No	703 (71.2)	469 (60.1)	1,172 (66.3)	
Unknown	197 (20.0)	263 (33.6)	460 (26.0)	
Left blank	8 (0.8)	11 (1.4)	19 (1.1)	
**Sexual orientation**				<0.001
Heterosexual	476 (48.2)	297 (38.0)	773 (43.7)	
Homosexual	33 (3.3)	21 (2.7)	54 (3.1)	
Bisexual	4 (0.4)	4 (0.5)	8 (0.5)	
Unknown/not applicable	277 (28.1)	327(41.9)	604 (34.2)	
Left blank	197 (20.0)	132 (16.9)	329 (18.5)	
**Gender identity**				<0.001
Transsexual man	1 (0.1)	3 (0.4)	4 (0.2)	
Transsexual woman	27 (2.7)	8 (1.0)	35 (2.0)	
Transvestite *(travesti)*	0 (0.0)	11 (1.4)	11 (0.6)	
Unknown/not applicable	762 (77.2)	627 (80.3)	1,389 (78.6)	

Regarding the variables related to the occurrence of violence, for both sexes, physical violence was the most frequently reported type, with 74.3% of cases in women and 73.3% in men, followed by psychological/ moral violence, with 44.1% of reported cases in females and 21.9% in males. As for the motivation for violence, for women the predominant reason was “unknown” (30.0%). However, among the reasons reported by them, sexism was the most frequent, i.e. 9.7%. Among men, following the largest proportion reported as “other” (36.4%), generational conflict (5.9%) and homelessness (5.5%) stood out. Work-related violence occurred mainly in the home setting in the case of women (27.9%) and in public thoroughfares for men (27.3%). For the variables presented in this table, the relationship with the victim’s sex was significant, except for the categories classified as “legal intervention” (p-value 0.118) and “other” (p-value 0.088) (Table 2).

Regarding the victim’s relationship/degree of kinship with the perpetrator of the violence, the most frequently reported perpetrator relationship among women was “stranger” (24.5%), cases involving “a person with an institutional relationship” (8,7%) and “boss” (7,3%) were also notable. For men, in addition to “stranger” (23.0%), “boss” was the second most frequent (21.1%). The probable perpetrator was most frequently male, both among women (59.8%) and men (77.0%). These perpetrator-related characteristics were significantly associated with victim sex (Table 3).

Regarding the temporal distribution of reported cases, an increase in reported cases of work-related violence was observed the 16 years of the series, rising from 16 cases in 2009 to 244 in 2024. Violence against women was predominant for most of the period, except in 2016, 2017, 2019 and 2023. In 2015, the reporting rate of work-related violence among women was 1.03/100,000 inhabitants, while among men it was 0.54/100,000 inhabitants (Table 4). The temporal trend analysis (2009-2024) showed a increasing reporting rate for both sexes, with 15.05% APC (95%CI 15.01;15.09) in women and 15.15% (95%CI 15.11;15.19) in men (Table 5).

**Table 2:** Frequency (n) and percentage (%) of reported cases of work-related violence according to sex and violence-related variables. Bahia, 2009-2024 (n=1,768)

Variables	Female	Male	Total	p-value
	(n=987; 55.8%)	(n=781; 44.2%)	(n=1,768; 100%)	
	n (%)	n (%)	n (%)	
**Type of violence**				
Physical	733 (74.3)	577 (73.9)	1,310 (74.1)	<0.001
Psychological/moral	435 (44.1)	171 (21.9)	606 (34.3)	<0.001
Torture	57 (5.8)	30 (3.8)	87 (4.9)	0.043
Sexual	184 (18.6)	3 (0.4)	187 (10.6)	<0.001
Human trafficking	12 (1.2)	88 (11.3)	100 (5.7)	<0.001
Financial/economic	68 (6.9)	18 (2.3)	86 (4.9)	<0.001
Negligence/abandonment	15 (1.5)	3 (0.4)	18 (1.0)	0.045
Legal intervention	12 (1.2)	3 (0.4)	15 (0.8)	0.118
Other	75 (7.6)	30(7.7)	135 (7.6)	0.088
**Motive for violence**				<0.001
Generational conflict	22 (2.2)	46 (5.9)	68 (3.8)	
Disability	14 (1.4)	5 (0.6)	19 (1.1)	
Homophobia/lesbophobia/biphobia/ transphobia	16 (1.6)	3 (0.4)	19 (1.1)	
Religious intolerance	2 (0.2)	1 (0.1)	3 (0.2)	
Racism	8 (0.8)	1 (0.1)	9 (0.5)	
Sexism	96 (9.7)	1 (0.1)	97 (5.5)	
Homelessness	23 (2.3)	43 (5.5)	66 (3.7)	
Xenophobia	0 (0.0)	2 (0.3)	2 (0.1)	
Other	216 (21.9)	284 (36.4)	500 (28.3)	
Unknown	296 (30.0)	196 (25.1)	492 (27.8)	
None of the above	294 (29.9)	199 (25.5)	493 (27.9)	
**Place of occurrence**				<0.001
Bar or similar	29 (2.9)	26 (3.3)	55 (3.1)	
Commerce/services	93 (9.4)	82 (10.5)	175 (9.9)	
School	32 (3.2)	9 (1.2)	41 (2.3)	
Shared housing	21 (2.1)	138 (17.7)	159 (9.0)	
Industries/construction	7 (0.7)	11 (1.4)	18 (1.0)	
Sports facility	6 (0.6)	3 (0.4)	9 (0.5)	
Home	275 (27.9)	108 (13.7)	383 (21.7)	
Public thoroughfare	165 (16.7)	213 (27.3)	378 (21.4)	
Other	255 (25.9)	138 (17.7)	393 (22.2)	
Unknown	101 (10.3)	53 (6.8)	154 (8.7)	
None of the above	3 (0.3)	0 (0.0)	3 (0.2)	

**Table 3:** Frequency (n) and percentage (%) of reported cases of work-related violence according to sex and variables related to the perpetrator of the violence. Bahia, 2009-2024 (n=1,768)

Variables	Female	Male	Total	p-value
	(n=987; 55.8%)	(n=781; 44.2%)	(n=1,768; 100%)	
	n (%)	n (%)	n (%)	
**Relationship/degree of kinship of the perpetrator**				<0.001
Mother/father	14 (1.4)	4 (0.5)	18 (1.0)	
Stepmother/stepfather	3 (0.3)	1 (0.1)	4 (0.2)	
Spouse	82 (8.3)	5 (0.6)	87 (4.9)	
Former spouse	63 (6.4)	3 (0.4)	66 (3.7)	
Boyfriend/girlfriend	13 (1.3)	4 (0.5)	17 (1.0)	
Former boyfriend/girlfriend	7 (0.7)	0 (0.0)	7 (0.4)	
Sibling/son or daughter	23 (2.3)	10 (1.3)	33 (1.9)	
Stranger	242 (24.5)	180 (23.0)	422 (23.9)	
Acquaintance	116 (11.8)	112 (14.3)	228 (12.9)	
Caregiver	7 (0.7)	2 (0.3)	9 (0.5)	
Boss	72 (7.3)	165 (21.1)	237 (13.4)	
Police officer/law enforcement agent	11 (1.1)	13 (1.7)	24 (1.4)	
Person with institutional relationship	86 (8.7)	28 (3.6)	114 (6.4)	
Self-inflicted	73 (7.4)	88 (11.3)	161 (9.1)	
Other	136 (13.8)	63 (8.1)	199 (11.3)	
Left blank	39 (4.0)	103 (13.2)	142 (8.0)	
**Sex of the probable perpetrator of the violence**				<0.001
Female	221 (22.4)	38 (4.9)	259 (14.6)	
Male	590 (59.8)	601 (77.0)	1191 (67.4)	
Both sexes	23 (2.3)	8 (1.0)	31 (1.8)	
Unknown	144 (14.6)	123 (15.7)	267 (15.1)	
None of the above	9 (0.9)	11 (1.4)	20 (1.1)	

**Table 4:** Frequency (n) and reporting rate (per 100,000 inhabitants) of work-related violence cases according to sex and reporting year. Bahia, 2009-2024 (n=1,768)

Year	Female			Male		Total
	Cases n=987	Reporting rate	Cases n=781	Reporting rate	Cases n=1,768	Reporting rate
2009	8	0.12	5	0.10	13	0.11
2010	17	0.34	9	0.19	26	0.26
2011	27	0.56	9	0.32	36	0.44
2012	38	0.65	15	0.31	53	0.49
2013	50	0.95	50	0.97	100	0.96
2014	79	1.30	60	1.07	139	1.19
2015	65	1.03	31	0.54	96	0.79
2016	54	0.98	42	1.44	96	1.21
2017	54	1.05	45	1.21	99	1.13
2018	68	1.03	45	0.90	113	0.97
2019	90	1.47	51	1.61	141	1.54
2020	48	0.76	38	0.62	86	0.69
2021	53	0.85	30	0.76	83	0.81
2022	80	1.27	47	0.78	127	1.03
2023	115	1.68	225	3.35	340	2.48
2024	141	1.94	79	1.27	220	1.62

**Table 5:** Annual percentage change (APC) and 95% confidence intervals (95%CI) of reporting rates (per 100,000 inhabitants) of work-related violence according to sex. Bahia, 2009-2024 (n=1,768)

Sex	Prais-Winsten regression coefficients			Trend (interpretation)
	APC (%)	95%CI	p-value	
Female	15.05	15.01;15.09	<0.003	Rising
Male	15.15	15.11;15.19	<0.002	Rising
Total	14.48	14.44;14.52	<0.003	Rising

## Discussion

The findings revealed that work-related violence in the state of Bahia is a worrying reality, particularly among women, who accounted for the largest proportion of reported cases. Reported cases of work-related violence increased over time, and in most years women were the most affected. Regarding the sociodemographic characteristics of the victims, the main differences between sexes were related to age: young adults accounted for the largest proportion of cases among both sexes; regarding race/color, the overall predominance was mixed race, although Black individuals were also frequently represented among women. Regarding the circumstances surrounding violence, sexism was noted as an important motive among women, while generational conflict was the most significant factor among men. Incidents were more frequent at home for women and in public thoroughfares for men. The trend analysis of the reporting rate of this form of violence over the 16-year period showed an increase in both strata, i.e. female and male.

The increase in reported cases may reflect improvements in reporting practices, which may contribute to ensuring comprehensive and multidisciplinary care to individuals experiencing violence. Furthermore, interventions are needed in both professional training and institutional practices, since healthcare professionals may simultaneously occupy the roles of victims and perpetrators of violence [14].

Regarding the higher occurrence of workplace violence among women, the sexist and patriarchal structures underlying society contribute to gender inequalities, contributing to the persistence of discrimination and the extrapolation of violence against women to the occupational sphere [15]. Sex refers to biological, genetic, and morphological characteristics. Gender is a broader concept that encompasses different identities and is a social, cultural, and political construct related to socially assigned roles and behaviors [16,17]. Sex and gender are distinct but related concepts, as biological differences are frequently used to justify gender inequality [18]. Thus, this study aimed to demonstrate gender inequalities by analyzing differences between sexes.

Sexism, gender-based discrimination, and prejudice were the predominant motivations in cases of violence against women. These mechanisms particularly affect women by reinforcing unequal social positions. Thus, gender stereotypes extend into the workplace and reinforce inequalities between men and women [19]. In the state of Paraná, in 2018, it was observed that being a woman may increase vulnerability to workplace violence perpetrated against nurses in urgent care units [20].

In the period studied here (2009-2024), the age groups with the highest number of reported cases of workplace violence among females were young adults (18-39 years). In 2022, in Brazil as a whole, women in the 25-39 age group had the highest level of employment (73.5%) [21], which may explain their greater exposure to workplace violence. A global systematic review, with data from 2010 to 2022, found that the category of young people (<40 years) was associated with increased risk of physical violence, verbal abuse and sexual harassment among female workers [22].

This study found that mixed-race and Black individuals were more frequently represented among reported cases of work-related violence against women, highlighting the intersection between race/ skin color and violence. In this context, structural racism shapes social perceptions and influences institutional relationships, reproducing itself in various spaces, including services and professional training [23,24]. From a historical perspective, this aspect affirms the racialization of the world of work, where precarious and less valued positions are assigned to non-White women, who are more vulnerable to racial violence [25]. However, for Bahia, this finding should be interpreted with caution, as it is one of the Brazilian states with the largest proportions of Black people.

This analysis also showed that the home was the most frequently reported location of violence among females. In the municipality of Maringá, in the state of Paraná, data collected between 2015-2019 [26] revealed women engaged in home-based occupations represented the majority of violence victims (75%). During the COVID-19 pandemic (2020-2021), a study conducted in 30 countries found that women who worked from home were more likely to experience intimate partner violence compared to those who worked in the workplace (OR 1.40, 95%CI 1.12;1.74) [27].

Regarding the location of reported violence, “home” was the most frequently reported location, followed by “other”. This vague category may obscure formal and informal work spaces. Institutional recognition of the phenomenon is hindered, since the reporting form does not include “workplace” as an available option for reporting the location of violence.

For the other variables analyzed, no relevant differences were observed between sexes. For both women and men, most victims were reported as single, without disabilities, identified as heterosexual, and, among women, most were not pregnant (Table 1). For both sexes, physical violence, followed by psychological/moral violence, was the most frequently reported form (Table 2). Regarding the perpetrator of the violent act, findings were similar between sexes, with perpetrators most commonly reported as strangers and male (Table 3).

Regarding marital status, in the state of Paraná, between 2015 and 2019, out of 319 reports of women assisted by the Police Station for Women in Maringá, single women accounted for the largest percentage of victims (52%) [26]. In contrast to our findings, regarding sexual orientation, in Brazil as a whole, in 2019, the prevalence of workplace violence was lower among heterosexual individuals (10%) than among homosexuals (22%) [28].

This study identified physical violence as the the most frequently reported type in Bahia, followed by psychological/moral violence. Nationally, however, an integrative review covering the period 2014-2023, found that 83.3% of the publications reviewed identified psychological violence as the most common type in work environments [29]. Regarding perpetrators, our findings are consistent with previous studies. Globally, a 2024 study identified men as the majority of perpetrators in cases of workplace violence, especially when the victims are women, accounting for 70% of aggressors in Burkina Faso and 90% in the Ivory Coast. [3]. Also in our study, the most frequently reported victim-perpetrator relationship was categorized as “stranger”, which highlights the difficulty of formally identifying perpetrators. However, when the aggressor was identified, the “boss” category appeared prominently, but only among male victims. 

This study has limitations that should be considered when interpreting the results. Limitations were related to the use of secondary data and administrative records. For many variables, a high proportion of variables were classified as “unknown/not applicable”, “other” and “none of the above”. Furthermore, high levels of missing data for some variables limited the analysis; occupation, for example, even though relevant to the phenomenon of work-related violence, was not included for this reason.

Factors that may have affected data quality include: the victim’s vulnerable situation, the sensitivity of the data on the form, and the fact that the professionals responsible for completing the report may not always be adequately trained in this approach. It should also be noted that, since reporting rates were not estimated according to race/color, the findings regarding this variable can be explained by the fact that a large proportion of Bahia’s population is classified as mixed-race or Black. Thus, they may not represent meaningful differences between these groups. Despite the problems identified, this study highlighted the complexity of workplace violence suffered by women in the state of Bahia, including variables rarely explored in previous studies, such as gender identity and sexual orientation.

In conclusion, this study highlights the need to address work-related violence in the state of Bahia as a structural problem shaped by gender inequalities that disproportionately affects women. The findings have important implications for practice and underscore the need for effective and targeted interventions, such as promoting organizational cultures based on respect and implementing effective public policies for prevention and protection. Addressing power imbalances and increasing awareness of workplace violence are essential for ensuring safe and inclusive work environments for all workers.

## Data Availability

The database and analysis codes used in this research are available in the Figshare Repository: https://doi. org/10.6084/m9.figshare.29874269.
