# Supplementary material for: Work-related violence from a gender perspective: a cross-sectional study with temporal trend analysis, Bahia, Brazil, 2009-2024
Source: Epidemiol Serv Saude. 2026 Aug 18;35:e20251061. doi: 10.1590/S2237-96222026v35e20251061.en (PMC13686420; doi:10.1590/S2237-96222026v35e20251061.en)
Supplement: Translation

# Translation: Violência relacionada ao trabalho sob a perspectiva de gênero: estudo transversal com análise de tendência temporal, Bahia, 2009-2024

**DOI:** 10.1590/S2237-96222026v35e20251061.pt

**Authors:** Adriana Galdino, Anívia de Souza Amaral, Érika Maria Andrade Silva, Alice Leite Velôso, Tarsila Rios Cabral, Ive Caroline Almeida Xavier

**Affiliations:** 1 Universidade Estadual do Sudoeste da Bahia, Campus de Jequié, Jequié, BA, Brazil Universidade Estadual do Sudoeste da Bahia Universidade Estadual do Sudoeste da Bahia Campus de Jequié Jequié BA Brazil; 2 Secretaria Municipal de Saúde de Jequié, Jequié, BA, Brazil Secretaria Municipal de Saúde de Jequié Secretaria Municipal de Saúde de Jequié Jequié BA Brazil

**Keywords:** Violência no Trabalho, Perspectiva de Gênero, Sistemas de Informação em Saúde, Violência de Gênero, Saúde do Trabalhador

## Abstract

**Objetivo:**

Analisar os casos de violência relacionada ao trabalho sob a perspectiva de gênero, na Bahia, de 2009 a 2024, considerando aspectos sociodemográficos e eventos da violência.

**Métodos:**

Estudo descritivo, transversal, com análise de tendência temporal, conduzido com dados de violência relacionada ao trabalho obtidos do Sistema de Informação de Agravos de Notificação (Sinan), envolvendo indivíduos maiores de 18 anos. Estimaram-se frequências relativas, distribuições proporcionais, análises bivariadas entre as variáveis descritivas e o sexo (feminino e masculino) e de tendência temporal da taxa de notificação de casos.

**Resultados:**

Foram registrados 1.768 casos de violência relacionada ao trabalho na Bahia durante o período. Observou-se maior frequência entre as mulheres (55,8%). Para o sexo feminino, a violência prevaleceu na faixa etária de 18-29 anos (36,5%) e nas pardas (46,7%). Também foi motivada por sexismo (9,7%) e ocorreu em residência (27,9%). Para o sexo masculino, predominou na mesma faixa etária (35,0%) e nos pardos (49,2%), além de ter sido motivada por conflito geracional (5,9%) e ocorrido em via pública (27,3%). A análise de tendência revelou crescimento da taxa de notificação em ambos os sexos, com variação percentual anual (VPA) de 15,0% (intervalo de confiança de 95%, IC95% 15,01;15,09) em mulheres e 15,1% (IC95% 15,11;15,19) em homens.

**Conclusão:**

Mulheres sofrem desproporcionalmente mais violência relacionada ao trabalho na Bahia, o que evidencia a urgência em abordar esse fenômeno como um problema estrutural e de gênero. É fundamental o desenvolvimento de políticas efetivas de prevenção e combate a esse tipo de violência.

Aspectos éticos Esta pesquisa utilizou bancos de dados de domínio público e anonimizados.

## Introdução

A violência ocupacional é definida como um conjunto de comportamentos, práticas ou ameaças, ocorridos uma ou mais vezes, que acarretam ou visam resultar em danos físicos, psicológicos, sexuais ou econômicos [1]. O assédio moral é um exemplo de violência caracterizada por ações sistemáticas de humilhação e constrangimento praticadas de modo recorrente [2]. Mensurando o impacto desses fenômenos, a Organização Internacional do Trabalho, em sua primeira pesquisa global sobre violência e assédio no trabalho, revelou que mais de um em cada cinco trabalhadores já foi afetado por esses agravos durante a vida profissional [3].

A violência e o assédio em razão de gênero no ambiente laboral configuram manifestações de violência ocupacio-nal e, neste estudo, são tratados também como violência relacionada ao trabalho. Questões de gênero permeiam o mercado de trabalho, uma vez que, entendida como construção social baseada em diferenças percebidas entre os sexos, essa categoria estrutura relações de poder que hierarquizam homens em detrimento das mulheres em diferentes contextos [4]. Em diferentes esferas do ambiente ocupacional, observam-se desigualdades de oportunidades e salariais, além de situações de assédio moral, sexual e de violência relacionada ao gênero [5].

As relações de poder no ambiente ocupacional são complexas. As agressões não ocorrem isoladamente, mas interseccionadas com outros marcadores sociais, como classe, raça/cor, etnia, deficiência e religião [6]. Mulheres que vivenciam discriminação na vida pública e privada tendem a estar mais expostas à violência e ao assédio no trabalho [3], o que intensifica experiências de opressão e exclusão.

A violência relacionada ao trabalho deve ser compreen-dida considerando as implicações de gênero, pois impacta desproporcionalmente as mulheres, o que pode ser verifi-cado em diferentes atividades laborais. Na Islândia, dados de uma coorte nacional de mulheres, em 2019, apontaram que 75% (n=1.178) vivenciaram assédio sexual ou violência no local de trabalho em algum momento da vida [7]. Em Adis Abeba, capital da Etiópia, entre 827 trabalhadoras da construção civil, a magnitude da violência contra mulheres no local de trabalho foi de 71% em 2021 [8].

Considerando-se a área da saúde, em Diamantina, Minas Gerais, no ano de 2017, a prevalência da violência no tra-balho para o sexo feminino foi de 84%, entre trabalhadores da Secretaria de saúde [9]. Entre enfermeiros de hospitais de Campo Grande, Mato Grosso do Sul, observou-se que no período 2018-2019 mulheres sofreram 5,83 vezes mais violência verbal do que homens [10]. Já em um hospital de Itaituba, Pará, em 2021, 69% das mulheres profissionais da urgência e da emergência sofreram violência relacionada ao trabalho [11]. Em hospital universitário do Rio Grande do Norte, durante a pandemia de covid-19 em 2020, 80% das mulheres residentes multiprofissionais relataram violência física/verbal [12].

No Brasil, a violência é um importante problema de saúde pública, sendo a violência interpessoal e autoprovocada um agravo de notificação compulsória universal. Portanto, deve ser registrada por profissionais de saúde que atendam pessoas em situação de violência, independentemente do tipo de unidade [13]. Todavia, são restritas as pesquisas com dados sobre violência de gênero no ambiente laboral, especialmen-te em recortes regionais e com grandes proporções populacionais. Nesse contexto, o presente estudo contribui para o aprofundamento do debate sobre a violência laboral e pode subsidiar a formulação de políticas públicas e estratégias institucionais de enfrentamento. Objetivou-se analisar os casos de violência relacionada ao trabalho sob a perspectiva de gênero, na Bahia, de 2009 a 2024, considerando aspectos sociodemográficos e eventos da violência.

## Métodos

### Delineamento e contexto do estudo

Trata-se de estudo descritivo, transversal, com análise de tendência temporal, no qual foram analisados registros de violência relacionada ao trabalho sob a perspectiva de gênero. Para esta pesquisa, a perspectiva de gênero correspondeu à apresentação e à análise dos dados de forma estratificada por sexo feminino e masculino. Foram avaliados casos notificados de violência relacionada ao trabalho em pessoas ≥18 anos, residentes nos municípios da Bahia, ocorridos entre 2009 e 2024, intervalo com dados disponíveis para análise. A Bahia tem população estimada de 14.141.626 habitantes segundo o censo de 2022, dentre os quais 7.305.940 são mulheres (51,7%). O estado conta com 417 municípios, distribuídos em nove Núcleos Regionais de Saúde.

Ressalta-se que este estudo é produto do Programa de Educação para o Trabalho pela Saúde (PET-Saúde), financiado pelo Ministério da Saúde do Brasil. Em sua 11ª edição, o Programa abordou o tema Equidade, tendo como eixo estruturante a *Valorização das trabalhadoras e futuras trabalhadoras no âmbito do SUS, saúde mental e violências relacionadas ao trabalho na saúde*. As pesquisadoras deste estudo integram essa edição do PET, que, na ocasião, foi realizado a partir da parceria entre a Universidade Estadual do Sudoeste da Bahia e a Secretaria Municipal de Saúde de Jequié-Bahia.

### Fontes de dados

Os dados sobre registros de violência foram obtidos no Sistema de Informação de Agravos de Notificação (Sinan), a partir da Ficha de Investigação de Violência Interpessoal e Autoprovocada. Esse instrumento é utilizado nacionalmente para notificação de casos de violência doméstica/ intrafamiliar, sexual e autoprovocada, bem como para tráfico de pessoas, trabalho escravo, infantil, tortura, intervenção legal e violências homofóbicas, envolvendo mulheres e homens de todas as idades no Brasil [13]. Também foram coletados dados do portal do Instituto Brasileiro de Geografia e Estatística sobre quantitativos populacionais da Bahia, segundo sexo e ano.

### Critérios de elegibilidade

A ficha de violência interpessoal e autoprovocada conta com 69 campos com informações desde a identificação da vítima até os encaminhamentos. Na base recebida, em todos os casos o campo “violência relacionada ao trabalho” estava registrado como “1=sim”, totalizando 2.328 observações. Destas, foram excluídos 557 casos devido à idade ser menor que 18 anos e 3 por registro de sexo ignorado. Não foram realizadas imputações nem verificação de duplicidade, pois a base analisada foi da Diretoria de Vigilância Epidemiológica da Secretaria de Saúde da Bahia, considerando-se que a limpeza do banco é rotina do setor e foi previamente realizada.

### Variáveis

As variáveis selecionadas foram as de maior potencial descritivo, organizadas nas subdimensões:

variáveis sociodemográficas: • sexo (feminino; masculino);
  • faixa etária (em anos: 18-29; 30-39; 40-49;
  • 50-59; 60-69; >70);
  • raça/cor da pele (amarela; branca; indígena; parda; preta; ignorado/em branco);
  • escolaridade (analfabeto; ensino fundamental; médio; superior; ignorado/não se aplica; em branco);
  • situação conjugal (casado/união consensual; solteiro; viúvo; separado; ignorado/não se aplica; em branco);
  • deficiência (sim; não; ignorado; em branco);
  • orientação sexual (heterossexual; homossexual; bissexual; ignorado/não se aplica; em branco);
  • identidade de gênero (homem transexual; mulher transexual; travesti; ignorado/não se aplica; em branco);
  • gestação (sim; não; ignorado/não se aplica);
variáveis da violência: • tipo (física; psicológica/moral; tortura; sexual; tráfico de seres humanos; financeira/econômica; negligência/abandono; intervenção legal; outros);
  • motivação (conflito geracional; deficiência; homofobia/lesbofobia/bifobia/transfobia; intolerância religiosa; racismo; sexismo; situação de rua; xenofobia; outros; ignorado; nenhum dos anteriores);
  • local (bar ou similar; comércio/serviços; escola; habitação coletiva; indústrias/construção; local de prática esportiva; residência; via pública; outros; ignorado; nenhum dos anteriores);
variáveis do autor da violência: • vínculo/grau de parentesco (mãe/pai; madrasta/padrasto; cônjuge; ex-cônjuge; namorado; ex-namorado; irmão/filho; desconhecido; conhecido; cuidador; patrão; policial/agente da lei; pessoa com relação institucional; própria pessoa; outros; em branco);
  • sexo do provável autor (feminino; masculino; ambos; ignorado; nenhum dos anteriores).

De modo geral, mantiveram-se as categorias conforme a ficha, exceto para escolaridade (categorias agrupadas) e tipo de violência, em que “trabalho infantil” foi considerado inconsistente para a faixa etária e seus registros agregados a “outros”. Para todas as variáveis, “não se aplica” foi incorporado a “ignorado”, por indicarem ausência de informação válida.

Ressalta-se que, no período analisado, ocorreram alterações nos campos da ficha; contudo, foram consideradas metodologicamente não relevantes para a análise transversal.

### Análise estatística

Os dados descritivos foram apresentados em números absolutos e proporções, segundo sexo da vítima. Estimaram-se taxas de notificação de casos de violência relacionada ao trabalho por ano de ocorrência, calculadas pela razão entre número de casos e respectivas populações. A associação entre variáveis categóricas foi analisada pelo teste qui-quadrado de Pearson. Para tendência temporal, utilizou-se regressão de Prais-Winsten, com correção para autocorrelação de primeira ordem. A taxa de notificação foi transformada em logaritmo natural, considerando-se o ano como variável independente. A variação percentual anual (VPA) e seus respectivos intervalos de confiança de 95% (IC95%) foram então calculados. Admitiu-se a classificação da tendência como crescente quando o IC95% da VPA foi positivo; decrescente quando negativo; e estacionária quando incluiu o valor zero. O nível de significância adotado foi de 5% em todas as análises inferenciais. Os dados foram analisados no software R versão 4.3.2.

## Resultados

No período entre 2009 e 2024, foram registrados na Bahia 1.768 casos de violência relacionada ao trabalho em pessoas acima de 18 anos. Destes, 987 (55,8%) ocorreram contra mulheres. A faixa etária com maior quantidade de casos foi a de 18-29 anos tanto para sexo feminino (36,5%) quanto para o masculino (35,0%). Quanto à raça/cor, a mais afetada foi a parda: em mulheres (46,7%) e em homens (49,2%). 

Em relação à escolaridade, entre as informadas, preponderaram mulheres com ensino médio (24,0%) e homens com ensino fundamental (22,8%). Quanto à situação conjugal, mulheres solteiras foram as principais vítimas (44,4%); entre os homens, prevaleceu o registro “ignorado” (36,4%), seguido de solteiros (32,1%). Pessoas sem deficiência foram as mais afetadas, tanto em mulheres (71,2%) quanto em homens (60,1%). Ainda considerando essa variável, o transtorno mental foi o principal, representando 3,4% no sexo feminino e 2,6% no masculino. Quanto à orientação sexual, a mais relatada foi a heterossexual nos casos femininos (48,2%); nos masculinos, predominou o registro “ignorado/não se aplica” (41,9%), seguido de heterossexuais (38,0%). Sobre identidade de gênero, em ambos os sexos a opção “ignorado/não se aplica” foi a mais frequente; nas mulheres 77,2% e nos homens 80,3%. A maior parte das mulheres não estava gestante (54,3%). Na análise bivariada, todas as características sociodemográficas apresentaram significância estatística para a relação com o sexo das vítimas de violência relacionada ao trabalho (Tabela 1).

**Tabela 1:** Frequência (n) e percentual (%) dos casos notificados de violência relacionada ao trabalho segundo sexo e variáveis sociodemográficas. Bahia, 2009-2024 (n=1.768)

Variáveis	Sexo feminino	Sexo masculino	Total	p-value
	(n=987; 55,8%)	(n=781; 44,2%)	(n=1.768; 100%)	
	n (%)	n (%)	n (%)	
**Faixa etária (anos)**				<0.001
18-29	360 (36.5)	273 (35.0)	633 (35.8)	
30-39	315 (31.9)	198 (25.3)	513 (29.0)	
40-49	208 (21.1)	166 (21.3)	374 (21.2)	
50-59	75 (7.6)	79 (10.1)	154 (8.7)	
60-69	18 (1.8)	47 (6.0)	65 (3.7)	
>70	11 (1.1)	18 (2.3)	29 (1.6)	
**Raça/cor**				<0.001
Amarela	9 (0.9)	43 (5.5)	52 (2.9)	
Branca	120 (12.2)	70 (9.0)	190 (10.7)	
Indígena	3 (0.3)	2 (0.3)	5 (0.3)	
Parda	461(46.7)	384 (49.2)	845 (47.8)	
Preta	200 (20.3)	127 (16.2)	327 (18.5)	
Ignorado/em branco	194 (19.6)	155 (19.8)	349 (19.8)	
Escolaridade				<0.001
Analfabeto	7 (0.7)	16 (2.0)	23 (1.3)	
Ensino fundamental	168 (17.0)	178 (22.8)	346 (19.6)	
Ensino médio	237 (24.0)	107 (13.7)	344 (19.5)	
Ensino superior	159 (16.2)	37 (4.8)	196 (11.0)	
Ignorado/não se aplica	339 (34.3)	388 (49.7)	727 (41.1)	
Em branco	77 (7.8)	55 (7.0)	132 (7.5)	
**Situação conjugal**				<0.001
Casado/união consensual	266 (27.0)	209 (26.8)	475 (26.9)	
Solteiro	438 (44.4)	251 (32.1)	689 (39.0)	
Viúvo	20 (2.0)	7 (0.9)	27 (1.5)	
Separado	39 (4.0)	17 (2.1)	56 (3.2)	
Ignorado/não se aplica	193 (19.5)	284 (36.4)	477 (26.9)	
Em branco	31 (3.1)	13 (1.7)	44 (2.5)	
**Deficiência**				<0.001
Sim	79 (8.0)	38 (4.9)	117 (6.6)	
Deficiência mental	16 (1.6)	5 (0.6)	21 (1.2)	
Transtorno mental	34 (3.4)	20 (2.6)	54 (3.1)	
Transtorno comportamental	14 (1.4)	8 (1.0)	22 (1.2)	
Visual	4 (0.4)	3 (0.4)	7 (0.4)	
Auditiva	4 (0.4)	1(0.1)	5 (0.3)	
Outra	4 (0.4)	3 (0.4)	7 (0.4)	
Não	703 (71.2)	469 (60.1)	1,172 (66.3)	
Ignorado	197 (20.0)	263 (33.6)	460 (26.0)	
Em branco	8 (0.8)	11 (1.4)	19 (1.1)	
**Orientação sexual**				<0.001
Heterossexual	476 (48.2)	297 (38.0)	773 (43.7)	
Homossexual	33 (3.3)	21 (2.7)	54 (3.1)	
Bissexual	4 (0.4)	4 (0.5)	8 (0.5)	
Ignorado/não se aplica	277 (28.1)	327(41.9)	604 (34.2)	
Em branco	197 (20.0)	132 (16.9)	329 (18.5)	
**Identidade de gênero**				<0.001
Homem transexual	1 (0.1)	3 (0.4)	4 (0.2)	
Mulher transexual	27 (2.7)	8 (1.0)	35 (2.0)	
Travesti	0 (0.0)	11 (1.4)	11 (0.6)	
Ignorado/não se aplica	762 (77.2)	627 (80.3)	1,389 (78.6)	

No que tange às variáveis relativas à ocorrência da violência, para ambos os sexos, a mais frequente foi a violência física, com 74,3% de casos em mulheres e 73,9% em homens, seguida da psicológica/moral, com 44,1% de notificações no sexo feminino e 21,9% no masculino. Quanto à motivação da violência, para as mulheres predominou o registro “ignorado” (30,0%). Porém, entre as razões informadas por elas, o sexismo foi o mais incidente, 9,7%. Já entre os homens, após a maior proporção informada como “outros” (36,4%), destacaram-se conflito geracional (5,9%) e situação de rua (5,5%). As situações de violência relacionada ao trabalho ocorreram principalmente nas residências para mulheres (27,9%) e em via pública para homens (27,3%). Para as variáveis dessa tabela, a relação com o sexo da vítima foi significativa, exceto para os tipos de violência registrados como “intervenção legal” (p-valor 0,118) e “outros” (p-valor 0,088) (Tabela 2).

Quanto ao vínculo/grau de parentesco da vítima com o autor da violência, o mais frequente nos registros das mulheres foi “desconhecido” (24,5%), destacando-se também os casos provocados por “pessoa com relação institucional” (8,7%) e por “patrão” (7,3%). Para os homens, além de “desconhecido” (23,0%), “patrão” foi o segundo mais frequente (21,1%). O sexo do provável autor foi predominantemente o masculino tanto entre mulheres (59,8%) quanto entre homens (77,0%). Essas características relativas ao autor da violência apresentaram relação estatisticamente significante com o sexo da vítima (Tabela 3).

Em relação à distribuição temporal dos registros, observa-se aumento das notificações de violência relacionada ao trabalho na Bahia ao longo dos 16 anos da série, passando de 16 casos em 2009 para 244 em 2024. Observa-se preponderância da violência no sexo feminino na maior parte do período, exceto em 2016, 2017, 2019 e 2023. Em 2015, a taxa de notificação de violência relacionada ao trabalho nas mulheres foi de 1,03/100 mil habitantes, enquanto nos homens foi de 0,54/100 mil habitantes (Tabela 4). A análise de tendência temporal (2009-2024) mostrou taxa de notificação crescente para ambos os sexos, com VPA de 15,05% (IC95% 15,01;15,09) em mulheres e 15,15% (IC95% 15,11;15,19) em homens (Tabela 5).

**Tabela 2:** Frequência (n) e percentual (%) dos casos notificados de violência relacionada ao trabalho segundo sexo e variáveis relativas à violência. Bahia, 2009-2024 (n=1.768)

Variáveis	Sexo feminino	Sexo masculino	Total	p-valor
	(n=987; 55,8%)	(n=781; 44,2%)	(n=1.768; 100%)	
	n (%)	n (%)	n (%)	
**Tipo de violência**			
Física	733 (74,3)	577 (73,9)	1.310 (74,1)	<0,001
Psicológica/moral	435 (44,1)	171 (21,9)	606 (34,3)	<0,001
Tortura	57 (5,8)	30 (3,8)	87 (4,9)	0,043
Sexual	184 (18,6)	3 (0,4)	187 (10,6)	<0,001
Tráfico de seres humanos	12 (1,2)	88 (11,3)	100 (5,7)	<0,001
Financeira/econômica	68 (6,9)	18 (2,3)	86 (4,9)	<0,001
Negligência/abandono	15 (1,5)	3 (0,4)	18 (1,0)	0,045
Intervenção legal	12 (1,2)	3 (0,4)	15 (0,8)	0,118
Outros	75 (7,6)	30(7,7)	135 (7,6)	0,088
**Motivação da violência**				<0,001
Conflito geracional	22 (2,2)	46 (5,9)	68 (3,8)	
Deficiência	14 (1,4)	5 (0,6)	19 (1,1)	
Homofobia/lesbofobia/bifobia/transfobia	16 (1,6)	3 (0,4)	19 (1,1)	
Intolerância religiosa	2 (0,2)	1 (0,1)	3 (0,2)	
Racismo	8 (0,8)	1 (0,1)	9 (0,5)	
Sexismo	96 (9,7)	1 (0,1)	97 (5,5)	
Situação de rua	23 (2,3)	43 (5,5)	66 (3,7)	
Xenofobia	0 (0,0)	2 (0,3)	2 (0,1)	
Outros	216 (21,9)	284 (36,4)	500 (28,3)	
Ignorado	296 (30,0)	196 (25,1)	492 (27,8)	
Nenhum dos anteriores	294 (29,9)	199 (25,5)	493 (27,9)	
**Local de ocorrência**				<0,001
Bar ou similar	29 (2,9)	26 (3,3)	55 (3,1)	
Comércio/serviços	93 (9,4)	82 (10,5)	175 (9,9)	
Escola	32 (3,2)	9 (1,2)	41 (2,3)	
Habitação coletiva	21 (2,1)	138 (17,7)	159 (9,0)	
Indústrias/construção	7 (0,7)	11 (1,4)	18 (1,0)	
Local de prática esportiva	6 (0,6)	3 (0,4)	9 (0,5)	
Residência	275 (27,9)	108 (13,7)	383 (21,7)	
Via pública	165 (16,7)	213 (27,3)	378 (21,4)	
Outros	255 (25,9)	138 (17,7)	393 (22,2)	
Ignorado	101 (10,3)	53 (6,8)	154 (8,7)	
Nenhum dos anteriores	3 (0,3)	0 (0,0)	3 (0,2)	

**Tabela 3:** Frequência (n) e percentual (%) dos casos notificados de violência relacionada ao trabalho segundo sexo e variáveis relacionadas ao autor da violência. Bahia, 2009-2024 (n=1.768)

Variáveis	Sexo feminino	Sexo masculino	Total	p-valor
	(n=987; 55,8%)	(n=781; 44,2%)	(n=1.768; 100%)	
	n (%)	n (%)	n (%)	
**Vínculo/grau de parentesco do autor**				<0,001
Mãe/pai	14 (1,4)	4 (0,5)	18 (1,0)	
Madrasta/padrasto	3 (0,3)	1 (0,1)	4 (0,2)	
Cônjuge	82 (8,3)	5 (0,6)	87 (4,9)	
Ex-cônjuge	63 (6,4)	3 (0,4)	66 (3,7)	
Namorado	13 (1,3)	4 (0,5)	17 (1,0)	
Ex-namorado	7 (0,7)	0 (0,0)	7 (0,4)	
Irmão/filho	23 (2,3)	10 (1,3)	33 (1,9)	
Desconhecido	242 (24,5)	180 (23,0)	422 (23,9)	
Conhecido	116 (11,8)	112 (14,3)	228 (12,9)	
Cuidador	7 (0,7)	2 (0,3)	9 (0,5)	
Patrão	72 (7,3)	165 (21,1)	237 (13,4)	
Policial/agente da lei	11 (1,1)	13 (1,7)	24 (1,4)	
Pessoa com relação institucional	86 (8,7)	28 (3,6)	114 (6,4)	
Própria pessoa	73 (7,4)	88 (11,3)	161 (9,1)	
Outros	136 (13,8)	63 (8,1)	199 (11,3)	
Em branco	39 (4,0)	103 (13,2)	142 (8,0)	
**Sexo do provável autor da violência**				<0,001
Feminino	221 (22,4)	38 (4,9)	259 (14,6)	
Masculino	590 (59,8)	601 (77,0)	1191 (67,4)	
Ambos os sexos	23 (2,3)	8 (1,0)	31 (1,8)	
Ignorado	144 (14,6)	123 (15,7)	267 (15,1)	
Nenhum dos anteriores	9 (0,9)	11 (1,4)	20 (1,1)	

**Tabela 4:** Frequência (n) e taxa de notificação (por 100 mil habitantes) dos casos de violência relacionada ao trabalho segundo sexo e ano de notificação. Bahia, 2009-2024 (n=1.768)

Ano	Sexo feminino		Sexo masculino		Total	
	Casos n=987	Taxa de notificação	Casos n=781	Taxa de notificação	Casos n=1.768	Taxa de notificação
2009	8	0,12	5	0,10	13	0,11
2010	17	0,34	9	0,19	26	0,26
2011	27	0,56	9	0,32	36	0,44
2012	38	0,65	15	0,31	53	0,49
2013	50	0,95	50	0,97	100	0,96
2014	79	1,30	60	1,07	139	1,19
2015	65	1,03	31	0,54	96	0,79
2016	54	0,98	42	1,44	96	1,21
2017	54	1,05	45	1,21	99	1,13
2018	68	1,03	45	0,90	113	0,97
2019	90	1,47	51	1,61	141	1,54
2020	48	0,76	38	0,62	86	0,69
2021	53	0,85	30	0,76	83	0,81
2022	80	1,27	47	0,78	127	1,03
2023	115	1,68	225	3,35	340	2,48
2024	141	1,94	79	1,27	220	1,62

**Tabela 5:** Variação percentual anual (VPA) e intervalos de confiança de 95% (IC95%) das taxas de notificação (por 100 mil habitantes) de violência relacionada ao trabalho segundo sexo. Bahia, 2009-2024 (n=1.768)

Sexo	Coeficientes da regressão de Prais-Winsten			Tendência (interpretação)
	VPA (%)	IC95%	p-valor	
Feminino	15,05	15,01;15,09	<0,003	Crescente
Masculino	15,15	15,11;15,19	<0,002	Crescente
Total	14,48	14,44;14,52	<0,003	Crescente

## Discussão

Os achados revelaram que a violência relacionada ao trabalho no estado da Bahia é uma realidade preocupante, especialmente para o sexo feminino, que concentra a maior proporção de casos quando comparada ao masculino. Observou-se aumento dos registros desse agravo ao longo do tempo, e na maioria dos anos as mulheres foram as mais afetadas. No que se refere às características sociodemográficas das vítimas, as diferenças mais relevantes entre os sexos foram relativas à idade: preponderaram mulheres adultas jovens e homens jovens; quanto à raça/cor, a predominância geral foi parda, porém, entre as mulheres destacou-se também a preta. Sobre as circunstâncias da violência, entre as mulheres notou-se o sexismo como motivo importante, enquanto nos homens foi o conflito geracional. As ocorrências foram mais frequentes nas residências para o sexo feminino e na via pública para o masculino. A análise de tendência da taxa de notificação desta violência para o período de 16 anos da série mostrou-se crescente em ambos os estratos, feminino e masculino.

O aumento dos casos de violência relacionada ao trabalho pode refletir o desenvolvimento progressivo das estratégias de notificação, que representa um passo importante para assegurar assistência integral e multiprofissional às pessoas que se encontram em situações de violência. Além disso, intervenções são necessárias tanto na formação quanto na prática institucional, uma vez que profissionais de unidades de saúde podem ocupar simultaneamente os papéis de vítimas e agentes perpetuadores da violência [14].

Sobre a preponderância de casos de violência laboral em mulheres, entende-se que o histórico machista e patriarcal da construção da sociedade sustenta desigualdades de gênero, motivando a persistência da discriminação e a extrapolação da violência contra mulheres para a esfera ocupacional [15]. Sexo refere-se às características biológicas, genéticas e morfológicas dos seres vivos. O gênero, categoria mais ampla, engloba diferentes identidades, sendo uma construção social, cultural e política relacionada aos papéis e comportamentos atribuídos socialmente a homens e mulheres [16,17]. Sexo e gênero são conceitos distintos, porém relacionados, pois diferenças biológicas são frequentemente utili-zadas para justificar a desigualdade de gênero [18]. Assim, este estudo buscou, por meio das diferenças entre o sexo, demonstrar desigualdades de gênero na Bahia para o fenômeno estudado.

O sexismo, discriminação ou preconceito baseado no sexo ou no gênero, foi a motivação predominante nos casos de violência contra mulheres. Ele as afeta especialmente ao colocá-las em posições de inferioridade na sociedade. Assim, estereótipos de gênero alcançam o âmbito laboral e reforçam desigualdades entre homens e mulheres [19]. No Paraná, em 2018, observou-se que o fato de ser mulher contribui para intensificar a violência ocupacional perpetrada contra enfermeiras em unidades de pronto atendimento [20].

No período aqui estudado (2009-2024), as idades com maior quantidade de ocorrências de violência laboral no sexo feminino foram das adultas-jovens (18-39 anos). Em 2022, no Brasil, as mulheres do grupo etário de 25 a 39 anos apresentaram o mais alto nível de ocupação (73,5%) [21], o que pode justificar maior exposição desse grupo à violência laboral. Em revisão sistemática realizada globalmente, com dados de 2010 a 2022, a categoria jovem (<40 anos) foi associada a risco aumentado de violência física, abuso verbal e assédio sexual nas trabalhadoras [22].

Observou-se neste estudo que as populações parda e preta aparecem com maior frequência nos registros de violência relacionada ao trabalho ocorridos em mulheres, evidenciando a interseccionalidade entre raça/cor da pele e violência. Nesse viés, o racismo estrutural perpassa a concepção de mundo dos sujeitos e estrutura relações institucionais, reproduzindo-se em diversos espaços, inclusive nos serviços e na formação profissional [23,24]. Esse aspecto, sob o ponto de vista histórico, afirma a racialização do mundo do trabalho, em que postos precarizados e menos valorizados são destinados às mulheres não brancas, mais vulneráveis a violências raciais [25]. Todavia, para a Bahia, este achado precisa ser analisado com cuidado, por ser um dos estados brasileiros com maiores contingentes de população negra.

Nesta análise, verificou-se também que a residência foi o local onde a violência foi mais frequente para o sexo feminino. No município Maringá-PR, Brasil, dados coletados entre 2015-2019 [26] revelaram que mulhe-res com ocupação profissional do lar foram as principais vítimas de violência (75%). No período da pandemia de covid-19 (2020-2021), em 30 países verificou-se que mulheres que trabalhavam em casa tinham mais chances de sofrer violência por parceiro íntimo em comparação àquelas que trabalhavam presencialmente (OR 1,40, IC 95% 1,12;1,74) [27].

Neste estudo, após “residência”, predominou “outros” para o registro de locais onde a violência informada ocorreu. Essa alternativa pouco específica pode ocultar espaços laborais formais e informais. Dificulta-se o reconhecimento institucional do fenômeno, já que na ficha de notificação não existe a opção “local de trabalho” entre as possibilidades de informação sobre o espaço.

Para as demais variáveis analisadas aqui, não houve diferenças relevantes entre os sexos. Tanto para mulheres quanto para homens, as vítimas estavam registradas principalmente como solteiras, não eram pessoas com deficiência, identificavam-se como heterossexuais e, no caso das mulheres, não estavam gestantes (Tabela 1). Em ambos os sexos, a violência física, seguida da psicológica/moral, foi o tipo mais frequentemente notificado (Tabela 2). Quanto ao autor do ato violento, os achados também foram semelhantes entre os sexos, sendo mais comumente descritos como pessoas desconhecidas e do sexo masculino (Tabela 3).

Sobre a condição conjugal, no Paraná, entre 2015-2019, de 319 laudos de mulheres atendidas pela Delegacia da Mulher de Maringá, as solteiras corresponderam ao maior percentual de vítimas (52%) [26]. Diferentemente dos achados deste estudo, quanto à orientação sexual, no Brasil, em 2019, a prevalência de violência no trabalho em heterossexuais foi menor (10%) do que em homossexuais (22%) [28].

Este estudo identificou a violência física como a mais frequentemente registrada na Bahia, seguida da psicológica/moral. Nacionalmente, porém, de 2014 a 2023, observou-se que 83,3% das publicações referiram-se à violência psicológica como a mais praticada em ambientes laborais [29]. Sobre o agressor, os achados deste estudo se assemelham a outros. Identificou-se, mundialmente, em 2024, que homens compõem a maioria dos autores nos casos de violência no tra-balho, sobretudo quando as vítimas são mulheres, representando 70% dos agressores em Burkina Faso e 90% na Costa do Marfim. [3]. Também nesta análise, o vínculo mais frequente entre vítimas e agressores foi classificado como “desconhecido”, o que evidencia a dificuldade de identificação formal dos perpetradores. No entanto, quando o agressor foi identificado, a categoria “patrão” apareceu com destaque, mas apenas entre as vítimas do sexo masculino.

Este estudo apresentou limitações que devem ser consideradas na interpretação dos resultados. Encontraram-se dificuldades decorrentes do uso de dados secundários e registros administrativos. Havia, para muitas variáveis, elevadas proporções de campos informados como “ignorado/não se aplica”, “outros” e “nenhum dos anteriores”. Além disso, o alto percentual de incompletude para algumas variáveis prejudicou a análise; a ocupação, por exemplo, mesmo sendo relevante para o fenômeno da violência relacionada ao trabalho, não foi incorporada por essa razão.

Destacam-se como fatores que podem ter interferido na qualidade dos registros: o contexto de fragilidade da vítima, a sensibilidade dos dados da ficha e o fato de que nem sempre os profissionais que preenchem a notificação estão treinados para a abordagem adequada. Ressalta-se ainda que, como não foram estimadas as taxas de notificação segundo raça/cor da pele, os achados relativos a essa variável podem ser explicados pelo fato de o maior contingente populacional do estado estar classificado como pardo e preto. Assim, não denotariam diferença relevante entre esses estratos. Apesar dos problemas apontados, esta pesquisa revelou parte da complexidade da violência laboral sofrida por mulheres no estado da Bahia, incluindo variáveis pouco exploradas em outros estudos, como identidade de gênero e orientação sexual.

Em conclusão, a pesquisa evidencia a urgência de abordar a violência relacionada ao trabalho no estado da Bahia como problema estrutural e de gênero, que afeta desproporcionalmente as mulheres. Os achados têm implicações significativas para a prática e ressaltam a necessidade de intervenções robustas e direcionadas, como, por exemplo, promover a cultura organizacional de respeito e implementar políticas públicas eficazes de prevenção e proteção. A transformação das relações de poder e a conscientização sobre a violência no trabalho são essenciais para garantir ambiente seguro e inclusivo para todos os trabalhadores.

## Data Availability

O banco de dados e os códigos de análise utilizados na pesquisa estão disponíveis no Repositório Figshare https://doi.org/10.6084/m9.figshare.29874269.

## References

[1] 1 International Labour Organization. C190 - Violence and Harassment Convention [Internet]. Geneva: International Labour Organization, 2019 [19 set. 2025]. https://normlex.ilo.org/dyn/nrmlx_en/f?p=NORMLEXPUB:12100:0::NO::P12100_ILO_CODE:C190 .

[2] 2 BUENO, RC; VITALINO, GBS. Assédio moral no trabalho: características e consequências psicopatológicas no sujeito. Rev Myth. 2023;20(2):26-41.

[3] 3 International Labour Organization; Lloyd's Register Foundation. Experiences of violence and harassment at work: a global first survey [Internet]. Geneva: International Labour Organization, Lloyd's Register Foundation, 2022 [19 set. 2025]. 10.54394/IOAX8567 .

[4] 4 MEDEIROS, FSB; CAMPOS, SAP. As relações de gênero, os estereótipos e a violência simbólica no mercado de trabalho. Rev Adm IMED. 2020;10(1):127-144.

[5] 5 CORTEZ, PA; SOUZA, MVR; SALVADOR, AP; OLIVEIRA, LFA. Sexismo, misoginia e LGBTQfobia: desafios para promover o trabalho inclusivo no Brasil. Physis. 2019;29(4):e290414. Available from: 10.1590/S0103-73312019290414.

[6] 6 ASSIS, DNC. Interseccionalidades [Internet]. Salvador: UFBA, Instituto de Humanidades, Artes e Ciências; Superintendência de Educação a Distância, 2019 [19 set. 2025]. https://repositorio.ufba.br/handle/ri/30892 .

[7] 7 JONSDOTTIR, SD; HAUKSDOTTIR, A; ASPELUND, T; JAKOBSDOTTIR, J; RUNARSDOTTIR, H; GUDMUNDSDOTTIR, B et al. Risk factors for workplace sexual harassment and violence among a national cohort of women in Iceland: a cross-sectional study. Public Health. 2022;7(9):e763-e774. Available from: 10.1016/s2468-2667(22)00201-8.PMC944997736057275

[8] 8 ASEGU, KA; DESSIE, AM; TILAHUN, TD; DAGNEW, GW; ANLEY, DT. Violence against women and associated factors among female construction workers in Addis Ababa, Ethiopia. BMC Psychiatry. 2023;23(1):547. Available from: 10.1186/s12888-023-05002-5.PMC1038590637507686

[9] 9 SIMÕES, MRL; BARROSO, HH; DE-AZEVEDO, DSS; DUARTE, ACM; BARBOSA, REC; FONSECA, GC et al. Violência no trabalho entre trabalhadores municipais do setor saúde de Diamantina, MG, 2017. Rev Bras Med Trab. 2020;18(1):82-90. Available from: 10.5327/z1679443520200425.PMC741368032783008

[10] 10 CEBALLOS, JB; FROTA, OP; NUNES, HFSS; ÁVALOS, PL; KRÜGEL, CD; FERREIRA JÚNIOR, MA et al. Violência física e verbal contra enfermeiros da classificação de risco: características, fatores relacionados e consequências. Rev Bras Enferm. 2020;73(suppl 5):e20190882. Available from: 10.1590/0034-7167-2019-0882.33338160

[11] 11 SILVA, FB; SILVEIRA, EF; GEDRAT, DC. Violência sofrida no trabalho: um estudo com profissionais do setor de urgência e emergência de um hospital do norte do Brasil. Aletheia. 2021;54(2):67-81. Available from: 10.29327/226091.54.2-7.

[12] 12 DANTAS, ESO; MAGNAGO, C; SANTOS, J; ARAÚJO FILHO, JD; MEIRA, KC. Prevalência e fatores associados à violência no trabalho contra residentes multiprofissionais durante a pandemia. Saude Debate. 2023;47(136):184-199. Available from: 10.1590/0103-1104202313612.

[13] 13 Brasil. Ministério da Saúde. Portaria GM/MS nº 5.201, de 15 de agosto de 2024. Altera o Anexo 1 do Anexo V à Portaria de Consolidação MS nº 4, de 28 de setembro de 2017, para incluir novas doenças na Lista Nacional de Notificação Compulsória de doenças, agravos e eventos em de saúde pública, nos serviços de saúde públicos e privados em todo o território nacional [Internet]. Brasília: Diário Oficial da União, 2024 [19 set. 2025]. https://www.in.gov.br/en/web/dou/-/portaria-gm/ms-n-5.201-de-15-de-agosto-de-2024-579010765 .

[14] 14 FAGUNDES, PM. Estudo das notificações de violência interpessoal e autoprovocada (2015-2021) e desenvolvimento de uma ferramenta digital de apoio em Cruz Alta/RS [dissertação] [Internet]. Dissertação (Mestrado) - Universidade Federal do Rio Grande do Sul, Porto Alegre, 2024 [19 set. 2025]. http://hdl.handle.net/10183/279196 .

[15] 15 FERNANDES, MNF; BRITO, ES; SILVA, AG; PICCOLO, IB; SOUZA, JL; CUNHA, LP et al. Assédio, sexismo e desigualdade de gênero no ambiente de trabalho. Rev Eletronica Fac Direito Franca. 2019;14(2):237-253. Available from: 10.21207/1983.4225.615.

[16] 16 NASCIMENTO, MG; KOSMINSKY, M; CHI, M. Papel do gênero na percepção e expressão da dor: revisão integrativa. Braz J Pain. 2020;3(1):58-62. Available from: 10.5935/2595-0118.20200013.

[17] 17 OKA, M; LAURENTI, C. Entre sexo e gênero: um estudo bibliográfico-exploratório das ciências da saúde. Saúde Soc. 2018;27(1):238-251. Available from: 10.1590/S0104-12902018170524.

[18] 18 ALBUQUERQUE, AG; BRAZ DA SILVA, AMT. Gênero como objeto de pesquisa em Representações Sociais. Res Soc Dev. 2019;8(7):e19871120. Available from: 10.33448/rsd-v8i7.1120.

[19] 19 ANDRADE, RD; FERNANDES, SCS. Expressões do sexismo no ambiente de trabalho: revisão de escopo. Psicol Teor Prat. 2024;26(2):1-27. Available from: 10.5935/1980-6906/ePTPSP15648.pt.

[20] 20 OLIVEIRA, CS; GALDINO, MJQ; BARRETO, MFC; SCHOLZE, AR; GOMES, JRM; MARTINS, JT. Violência no trabalho: vivências e estratégias de enfrentamento por enfermeiros de unidades de pronto atendimento. Cienc Enferm. 2022;28:10. Available from: 10.29393/CE28-10VTCJ60010.

[21] 21 Brasil. Instituto Brasileiro de Geografia e Estatística. Pesquisa Nacional por Amostra de Domicílios Contínua: primeiros resultados do 1º trimestre de 2022 [Internet]. Rio de Janeiro: IBGE, 2022 [19 set. 2025]. https://ftp.ibge.gov.br/Trabalho_e_Rendimento/Pesquisa_Nacional_por_Amostra_de_Domicilios_continua/Trimestral/Fasciculos_Indicadores_IBGE/2022/pnadc_202201_trimestre_caderno.pdf .

[22] 22 AJUWA, MEP; VEYRIER, CA; CABROLIER, LC; CHASSANY, O; MARCELLIN, F; YAYA, I et al. Workplace violence against female healthcare workers: a systematic review and meta-analysis. BMJ Open. 2024;14(8):e079396. Available from: 10.1136/bmjopen-2023-079396.PMC1136978339209501

[23] 23 SILVA, LB; CAMPOS, DS; ARAÚJO, MVR; REIS, RS. "Mesmo que a gente seja a mão que cuida": médicas negras e racismo estrutural no contexto da atenção primária à saúde. Cien Saude Colet. 2024;29(3):e07622023. Available from: 10.1590/1413-81232024293.07622023.38451650

[24] 24 SHARIF, MZ; GARCÍA, JJ; MITCHELL, U; DELLOR, ED; BRADFORD, NJ; TRUONG, M. Racism and structural violence: interconnected threats to health equity. Front Public Health. 2022;9:676783. Available from: 10.3389/fpubh.2021.676783.PMC885029435186857

[25] 25 ANDRADE, CB. Trabalhadoras migrantes latino-americanas e violências: rumo a uma visão interseccional? Cien Saude Colet. 2023;28(11):3281-3287. Available from: 10.1590/1413-812320232811.16622022.37971010

[26] 26 AMARAL, MA; DULTRA, JCR; MACKINCS, GP; AMARAL, V. Perfil Epidemiológico da Violência contra a Mulher em um Município da Região Sul do Brasil. Arch Health Invest. 2022;11(4):599-604. Available from: 10.21270/archi.v11i4.5555.

[27] 27 MIALL, N; FRANCIS, SC; STÖCKL, H; TUCKER, JD. Working from home and intimate partner violence among cis-women during the COVID-19 pandemic: evidence from a global, cross-sectional study. BMC Public Health. 2023;23(1):965. Available from: 10.1186/s12889-023-15785-7.PMC1021431337237282

[28] 28 FRANCISCO, PMSB; ASSUMPÇÃO, D; BANDINI, M; LUCCA, SR. Prevalência de violência psicológica na população brasileira ocupada e sua ocorrência no ambiente de trabalho: Pesquisa Nacional de Saúde 2019. Rev Bras Epidemiol. 2025;28:e250025. Available from: 10.1590/1980-549720250025.2.PMC1212924140465874

[29] 29 BRITO, ARA; CONTRERA, L; QUEIROZ-CARDOSO, AI; CAVALCANTE, FM. Características epidemiológicas da violência relacionada ao trabalho no Brasil: revisão integrativa. Rev Enferm Atual In Derme. 2025;99(suppl.1):e025085. Available from: 10.31011/reaid-2025-v.99-n.supl.1-art.2536.

[30] 30 PET-Saúde, Equidade 11ª Edição UESB. Bancos e análise - Violência relacionada ao trabalho na Bahia, Brasil (2025) [dataset]. Figshare Dataset, 2025 [16 jun. 2025]. Available from: Available from: 10.6084/m9.figshare.29874269.v3 .
